# Benefits of glycopyrrolate/formoterol fumarate metered dose inhaler (GFF MDI) in improving lung function and reducing exacerbations in patients with moderate-to-very severe COPD: a pooled analysis of the PINNACLE studies

**DOI:** 10.1186/s12931-020-01388-y

**Published:** 2020-05-25

**Authors:** Fernando J. Martinez, Brian J. Lipworth, Klaus F. Rabe, David J. Collier, Gary T. Ferguson, Sanjay Sethi, Gregory J. Feldman, Gerald O’Brien, Martin Jenkins, Colin Reisner

**Affiliations:** 1 Weill Cornell Medical College, New York-Presbyterian Hospital/Weill Cornell Medical Center, 525 E 68th St, Room M-522, Box 130, New York, NY 10065 USA; 2 Scottish Centre for Respiratory Research, Ninewells Hospital, University of Dundee, Dundee, Scotland UK; 3 LungenClinic Grosshansdorf and Christian-Albrechts University Kiel, Airway Research Center North, Member of the German Center for Lung Research (DZL), Kiel, Germany; 4 William Harvey Research Institute, Barts and the London School of Medicine and Dentistry, Queen Mary University of London, London, UK; 5 Pulmonary Research Institute of Southeast Michigan, Farmington Hills, MI USA; 6 University at Buffalo, SUNY, Buffalo, NY USA; 7 S. Carolina Pharmaceutical Research, Spartanburg, SC USA; 8 formerly of AstraZeneca, Wilmington, DE USA; 9 AstraZeneca, Cambridge, UK; 10 AstraZeneca, Morristown, NJ USA

**Keywords:** Chronic obstructive pulmonary disease, Clinically important deterioration, Exacerbations, Fixed-dose combination, Formoterol fumarate dihydrate, GFF MDI, Glycopyrronium, LAMA/LABA, Metered dose inhaler, Symptomatic

## Abstract

**Background:**

The Phase III PINNACLE studies assessed the efficacy and safety of glycopyrrolate/formoterol fumarate metered dose inhaler (GFF MDI), a dual long-acting bronchodilator for chronic obstructive pulmonary disease (COPD). Here we present a pre-specified pooled analysis of PINNACLE-1, PINNACLE-2, and PINNACLE-4.

**Methods:**

PINNACLE-1, -2, and -4 were multicenter, double-blind, randomized controlled trials that enrolled patients with moderate-to-very severe COPD, with no requirement for exacerbation history or a high symptom burden. Patients received GFF MDI 18/9.6 μg, glycopyrrolate (GP) MDI 18 μg, formoterol fumarate (FF) MDI 9.6 μg, or placebo MDI, twice-daily for 24 weeks. The primary endpoint of the pooled analysis was the change from baseline in morning pre-dose trough forced expiratory volume in 1 s (FEV_1_) at week 24. Secondary endpoints included COPD exacerbations and clinically important deterioration (CID). Adverse events were also assessed.

**Results:**

The pooled intent-to-treat population included 4983 patients; of these, 61.9% had a COPD assessment test (CAT) score ≥15, and 25.0% had experienced ≥1 moderate/severe exacerbation in the past year. At week 24, GFF MDI improved morning pre-dose trough FEV_1_ versus GP MDI (least squares mean [LSM] difference [95% confidence interval (CI)]: 59 mL [43, 75]), FF MDI (65 mL [48, 81]), and placebo MDI (146 mL [125, 166]); all *p* < 0.0001. GFF MDI reduced the risk of a moderate/severe exacerbation by 18% (*p* = 0.0168), 15% (*p* = 0.0628), and 28% (*p* = 0.0012) compared with GP MDI, FF MDI, and placebo MDI, respectively. In general, exacerbation risk reduction with GFF MDI versus comparators was greater in subgroups of symptomatic patients (CAT ≥15) and those who had an exacerbation history, than in the pooled intent-to-treat population. The risk of CID was also lower with GFF MDI versus GP MDI (23% decrease), FF MDI (17%), and placebo MDI (49%); all *p* < 0.0001. All treatments were well tolerated, with no unexpected safety signals.

**Conclusions:**

This pooled analysis of the PINNACLE studies demonstrated that GFF MDI improved lung function and reduced the risk of exacerbations compared with monocomponents and placebo in patients with COPD. Exacerbation reductions with GFF MDI versus comparators were generally greater in patients with higher symptom burden and those with exacerbation history.

**Trial registration:**

ClinicalTrials.gov NCT01854645, NCT01854658, and NCT02343458. Registered 13 May 2013 (NCT01854645, NCT01854658) and 6 January 2015 (NCT02343458).

## Introduction

Reducing the future risk of chronic obstructive pulmonary disease (COPD) exacerbations is one of the main goals for the treatment of the disease [1]. Exacerbations cause a substantial impact on patients’ lives; they are associated with a greater rate of lung function decline [2] and worse quality of life [3] compared with patients who do not experience exacerbations. The Global Initiative for Chronic Obstructive Lung Disease (GOLD) 2020 report recommends long-acting muscarinic antagonist (LAMA)/long-acting β_2_-agonist (LABA) combinations for prevention of exacerbations in some patients, prior to escalation to triple therapy [1]. However, there are limited data regarding the benefit of LAMA/LABA fixed-dose combinations (FDCs) versus the corresponding monocomponents in reducing exacerbations in patients with COPD [4–6].

Glycopyrrolate/formoterol fumarate metered dose inhaler 18/9.6 μg (GFF MDI; Bevespi Aerosphere®), an FDC of the LAMA glycopyrrolate and the LABA formoterol fumarate, delivered using innovative co-suspension delivery technology, is approved as a maintenance treatment for COPD. The efficacy and safety of GFF MDI compared with its respective monocomponents have been demonstrated in patients with moderate-to-very severe COPD in the pivotal Phase III studies PINNACLE-1, PINNACLE-2, and PINNACLE-4 (NCT01854645, NCT01854658, and NCT02343458; all 24 weeks), and PINNACLE-3 (NCT01970878; 28-week safety extension study of PINNACLE-1 and -2), in patients from the USA, Asia, Europe, Australia, and New Zealand [7–9]. However, the individual pivotal studies, which were not enriched for patients with a high risk of exacerbations, were not sufficiently powered to interrogate the impact of GFF MDI on exacerbations. Yet, a pooled analysis of these studies provides a reasonably large body of evidence in which to assess the exacerbation benefits of GFF MDI.

The objective of this pre-specified, integrated analysis of individual patient data from the three 24-week studies, PINNACLE-1, -2, and -4, was to further evaluate the efficacy of GFF MDI, by using the combined data set to provide a more precise estimation of the treatment effects on lung function, COPD exacerbations, and clinically important deterioration (CID) endpoints in the pooled population, and to evaluate safety in a large patient population.

## Methods

### Study design, patients, and treatments

**Fig. 1:**
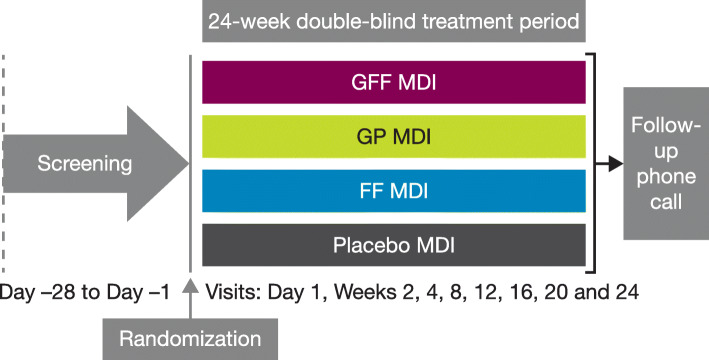
PINNACLE-1, -2, and -4 study design. PINNACLE-1 also included an open-label tiotropium arm (not shown). FF, formoterol fumarate; GFF, glycopyrrolate/formoterol fumarate; GP, glycopyrrolate; MDI, metered dose inhaler

Study designs, eligibility criteria, treatments, and statistical methods for the studies included in this analysis have been reported previously [7, 9]. In brief, PINNACLE-1, -2, and -4 were three Phase III multicenter, randomized, double-blind, parallel-group, placebo-controlled trials (Fig. 1). PINNACLE-1 was conducted across sites in the USA, Australia, and New Zealand; PINNACLE-2 encompassed sites in the USA; and PINNACLE-4 included sites across Asia, Europe, and the USA. Each study enrolled patients who were 40–80 years of age and current or ex-smokers (history of ≥10 pack-years), with moderate-to-very severe COPD, defined by a post-bronchodilator forced expiratory volume in 1 s (FEV_1_)/forced vital capacity (FVC) ratio < 0.7, and a post-bronchodilator FEV_1_ < 80% predicted normal value at screening [1]. Patients with FEV_1_ < 30% predicted normal (i.e. very severe airflow limitation) were required to have a post-bronchodilator FEV_1_ ≥ 750 mL. There was no exacerbation history requirement or minimum symptom score for study entry, in contrast to several randomized controlled trials of other LAMA/LABA combinations that enrolled entirely symptomatic populations, e.g. patients with a modified Medical Research Council dyspnea scale (mMRC) score ≥2 [10], or those meeting a symptom diary score threshold [11].

Exclusion criteria included a current diagnosis of asthma, concomitant respiratory disorders including, but not limited to, lung cancer and alpha-1 antitrypsin deficiency, or any underlying renal, liver, endocrine, cardiac, or other medical condition that, in the opinion of the investigator, may have influenced the study results or the patient’s ability to participate. Patients were randomized to 24 weeks’ treatment with GFF MDI 18/9.6 μg (also known as, and equivalent to, glycopyrronium/formoterol fumarate dihydrate 14.4/10.0 μg), glycopyrrolate (GP) MDI 18 μg, formoterol fumarate (FF) MDI 9.6 μg, or matched placebo MDI (all administered as two actuations twice daily). Also, the PINNACLE-1 study included an open-label tiotropium arm (one capsule inhaled once daily) as an active control group, but this was not included in the pooled analyses as it was only assessed in one of the three studies.

Patients using an inhaled corticosteroid (ICS) as part of their maintenance therapy at baseline were permitted to continue with the ICS component, providing they had been on a stable dose for ≥4 weeks prior to screening. Sponsor-provided albuterol sulfate (Ventolin HFA) was permitted as rescue medication throughout the studies. The PINNACLE studies were conducted in accordance with Good Clinical Practice, including the International Conference on Harmonisation guidelines and the Declaration of Helsinki. All patients provided written informed consent prior to screening.

### Outcomes included in the pooled analysis

The change from baseline in morning pre-dose trough FEV_1_ at week 24 (for the US statistical analysis approach) or over 24 weeks (EU approach) was the primary or co-primary endpoint of each study and also the primary endpoint of the pooled analysis. Secondary endpoints of the pooled analysis included peak change from baseline in FEV_1_ within 2 h post-dose, rate of moderate or severe COPD exacerbations, time to the first moderate or severe COPD exacerbation, time to treatment failure, and time to first CID. A moderate exacerbation was defined as an exacerbation that required treatment with systemic corticosteroids and/or antibiotics, and a severe exacerbation was defined as one that resulted in hospitalization or death. Treatment failure was defined as a moderate or severe COPD exacerbation or discontinuation from study for any reason. A CID was defined as a ≥100 mL reduction in morning pre-dose trough FEV_1_ and/or a worsening (increase) of ≥4 units in St George’s Respiratory Questionnaire (SGRQ) at any post-baseline study visit, and/or the occurrence of a treatment-emergent moderate or severe COPD exacerbation. While we report both the rate and the time to the first moderate or severe COPD exacerbation here, the time to first exacerbation may provide a better estimation of the treatment benefits compared with the rate of moderate or severe exacerbations, as patients with a severe COPD exacerbation or >2 moderate COPD exacerbations were discontinued from the PINNACLE studies. Pooled safety analyses included adverse events and serious adverse events.

### Statistical analysis

The statistical analysis plan for the pooled analyses of PINNACLE-1, -2, and -4 was developed prior to the unblinding of PINNACLE-4 and was based upon an update to a pre-specified analysis plan written prior to the unblinding of PINNACLE-1 and -2. Pooled analyses of spirometry, exacerbations, and treatment failure were included in the original analysis plan, with the COPD assessment test (CAT) ≥15 subgroup and CID analyses defined in the pooled analysis plan prior to the unblinding of PINNACLE-4. The pooled intent-to-treat (ITT) and safety populations included patients who were randomized to treatment and received any study treatment in PINNACLE-1, -2, or -4. Patients who received open-label tiotropium in PINNACLE-1 were excluded from both the ITT and safety populations as there was no tiotropium arm in the other PINNACLE studies. There was no control for multiplicity for the pooled data as this had been performed in the individual studies.

Twenty-two patients participated at multiple sites within a study, or in more than one of the PINNACLE studies. These patients were excluded from both the pooled ITT and safety populations if they had overlapping treatment exposure. If exposure did not overlap, data from their first exposure were included in both populations, while subsequent exposure data were included only in the safety population.

In addition to the pooled ITT population, we report results for exacerbation, treatment failure, and CID endpoints from subgroups of patients who were symptomatic (defined as a CAT score ≥15 at baseline) or with a history of exacerbations (defined as ≥1 moderate or severe exacerbations in the year prior to the baseline visit). The CAT ≥15 subgroup was pre-defined as the symptomatic population in the pooled analysis plan based on findings from PINNACLE-1 and -2 [6], while the analyses in the exacerbation history subgroup were produced post-hoc, to understand the consistency of the findings.

The change from baseline in morning pre-dose trough FEV_1_ and peak change from baseline in FEV_1_ within 2 h post-dose were analyzed using a linear repeated measures model, with baseline FEV_1_, percent reversibility to albuterol sulfate, study (PINNACLE-1/PINNACLE-2/PINNACLE-4), treatment, visit, and treatment by visit interaction as covariates. The time to first COPD exacerbation, time to treatment failure, and time to first CID were analyzed using Cox regression models that adjusted for baseline percent predicted FEV_1_, study, baseline CAT score, baseline COPD exacerbation history (yes/no), smoking status at baseline (former/current), baseline continuous eosinophil count, and ICS use at baseline (yes/no). The rate of moderate or severe COPD exacerbations was analyzed using negative binomial regression, adjusting for the same covariates, with treatment exposure used as an offset variable. Patients who did not experience a COPD exacerbation were censored at week 24, and those who withdrew from the study without experiencing a COPD exacerbation were censored at the date of withdrawal.

## Results

### Patient population

**Fig. 2:**
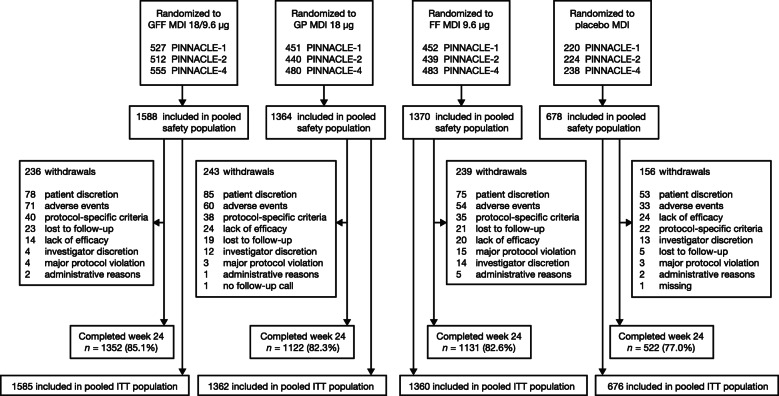
Patient disposition (pooled PINNACLE population). Patients were excluded from the pooled population if they did not receive any study treatment or if they had overlapping treatment exposure from enrollment in more than one PINNACLE study (further details are provided in the Methods). FF, formoterol fumarate; GFF, glycopyrrolate/formoterol fumarate; GP, glycopyrrolate; ITT, intent-to-treat; MDI, metered dose inhaler.

A total of 5000 patients were included in the pooled safety population (Fig. 2). Of these, 4983 were included in the pooled ITT population.

Baseline demographics and clinical characteristics were generally similar across the ITT populations of all three studies, with some differences observed in race, gender, body mass index, smoking status, and symptom burden (as measured by CAT score), mainly due to the inclusion of Asian patients in PINNACLE-4 [7, 9]. Most of the Asian patients recruited in PINNACLE-4 were male, and had, on average, a lower baseline CAT score than patients from other regions. Accordingly, the proportion of male patients in PINNACLE-4 (74.1%) was higher than in PINNACLE-1 and -2 (56.4 and 55.1%, respectively), and fewer patients in PINNACLE-4 had CAT scores ≥15 compared with PINNACLE-1 or -2 (48.3% vs. 68.2 and 69.5%, respectively). A greater proportion of patients in PINNACLE-1 and -2 were current smokers (54.1 and 53.2%, respectively) than in PINNACLE-4 (44.9%).

Baseline characteristics for the pooled ITT population, symptomatic, and exacerbation history subgroups are shown in Table 1. As expected, a larger proportion of patients in the exacerbation history subgroup reported ICS use at screening (47.3%) compared with the pooled ITT population and symptomatic subgroup (34.6–35.8%). Within the pooled ITT population and the symptomatic and exacerbation history subgroups, baseline demographics were generally similar across treatment groups (data not shown).

**Table 1:** Patient demographics and clinical characteristics (pooled ITT, exacerbation history, and CAT ≥15 populations)

	Pooled ITT (*n* = 4983)	Exac. history^a^ (*n* = 1246)	CAT ≥15 (*n* = 3082)
Mean age, years (SD)	63.3 (8.1)	62.8 (8.1)	62.1 (8.2)
Age ≥65 years, *n* (%)	2339 (46.9)	551 (44.2)	1243 (40.3)
Male, *n* (%)	3086 (61.9)	705 (56.6)	1774 (57.6)
Race, *n* (%)			
White	3944 (79.1)	958 (76.9)	2653 (86.1)
Asian	711 (14.3)	203 (16.3)	192 (6.2)
Black	302 (6.1)	80 (6.4)	218 (7.1)
Other	26 (0.5)	5 (0.4)	19 (0.6)
Current smoker, *n* (%)	2528 (50.7)	588 (47.2)	1824 (59.2)
Mean number of pack-years smoked^b^ (SD)	49.3 (26.1)	48.6 (25.8)	50.7 (26.3)
Mean number of years smoked (SD)	39.4 (10.3)	38.8 (10.3)	40.2 (9.9)
Mean total CAT score^c^ (SD)	17.3 (7.5)	18.1 (7.6)	22.0 (5.2)
CAT ≥10, *n* (%)	4166 (83.6)	1064 (85.4)	3082 (100.0)
CAT ≥15, *n* (%)	3082 (61.9)	823 (66.1)	3082 (100.0)
CAT ≥20, *n* (%)	1879 (37.7)	519 (41.7)	1879 (61.0)
ICS use at baseline, *n* (%)	1723 (34.6)	589 (47.3)	1104 (35.8)
Exacerbation history in the past year, *n* (%)			
≥1 moderate or severe exacerbation	1246 (25.0)	1246 (100.0)	823 (26.7)
≥1 severe exacerbation	302 (6.1)	302 (24.2)	211 (6.8)
COPD severity, *n* (%)			
Mild^d^	30 (0.6)	6 (0.5)	8 (0.3)
Moderate	2764 (55.5)	619 (49.7)	1558 (50.6)
Severe	1971 (39.6)	551 (44.2)	1344 (43.6)
Very severe	218 (4.4)	70 (5.6)	172 (5.6)
GOLD 2017 Category, *n* (%)			
A	729 (14.6)	105 (8.4)	0
B	3693 (74.1)	591 (47.4)	2705 (87.8)
C	73 (1.5)	73 (5.9)	0
D	473 (9.5)	473 (38.0)	377 (12.2)
Missing	15 (0.3)	4 (0.3)	0
Mean COPD duration, years^e^ (SD)	7.2 (6.3)	7.4 (6.1)	7.6 (6.4)
Mean pre-bronchodilator FEV_1_, % predicted (SD)	45.1 (13.8)	43.2 (13.6)	43.5 (13.7)
Mean post-bronchodilator FEV_1_, % predicted (SD)	52.5 (13.9)^f^	50.8 (14.0)	50.9 (13.9)
Mean pre-bronchodilator FVC, % predicted (SD)	71.2 (16.2)	70.0 (15.9)	68.6 (15.7)
Mean post-bronchodilator FVC, % predicted (SD)	80.0 (16.3)^f^	79.5 (16.3)	77.5 (15.8)

^a^The exacerbation history population includes patients with ≥1 moderate or severe exacerbation in the previous year

^b^Pack-years smoked = (number of cigarettes per day/20)* number of years smoked

^c^*n* = 4968 (pooled ITT), *n* = 1242 (Exac. history)

^d^Characterized as mild COPD due to the application of an Asian correction factor in PINNACLE-4, and were identified as protocol deviations

^e^*n* = 4970 (pooled ITT), *n* = 1243 (Exac. history), *n* = 3076 (CAT ≥15)

^f^*n* = 4981

*CAT* COPD assessment test, *COPD* chronic obstructive pulmonary disease, *Exac*. exacerbation, *FEV*_*1*_ forced expiratory volume in 1 s, *FVC* forced vital capacity, *GOLD* Global Initiative for Chronic Obstructive Lung Disease, *ICS* inhaled corticosteroid, *ITT* intent-to-treat, *SD* standard deviation

### Efficacy

#### Lung function

**Fig. 3:**
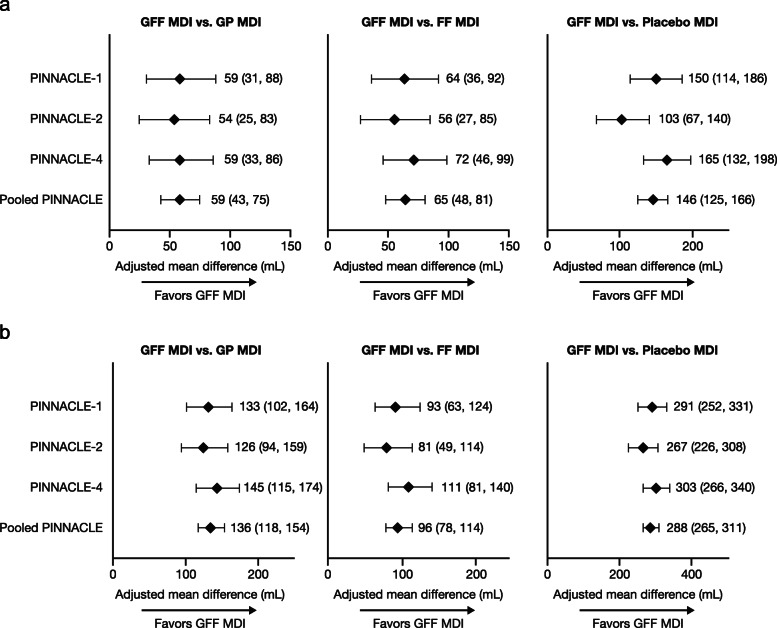
Lung function endpoints at week 24. Change from baseline in morning pre-dose trough FEV_1_ (**a**) and peak change from baseline in FEV_1_ within 2 h post-dose (**b**) (ITT population of the individual and pooled studies). Data are least squares mean treatment differences (95% confidence intervals) shown in mL. *p* < 0.0001 for all comparisons except for trough FEV_1_ in PINNACLE-2, GFF MDI versus GP MDI (*p* = 0.0003) and FF MDI (*p* = 0.0002). Data for PINNACLE-1, -2, and -4 studies have been published previously [7, 9]. FEV_1_, forced expiratory volume in 1 s; FF, formoterol fumarate; GFF, glycopyrrolate/formoterol fumarate; GP, glycopyrrolate; ITT, intent-to-treat; MDI, metered dose inhaler

**Fig. 4:**
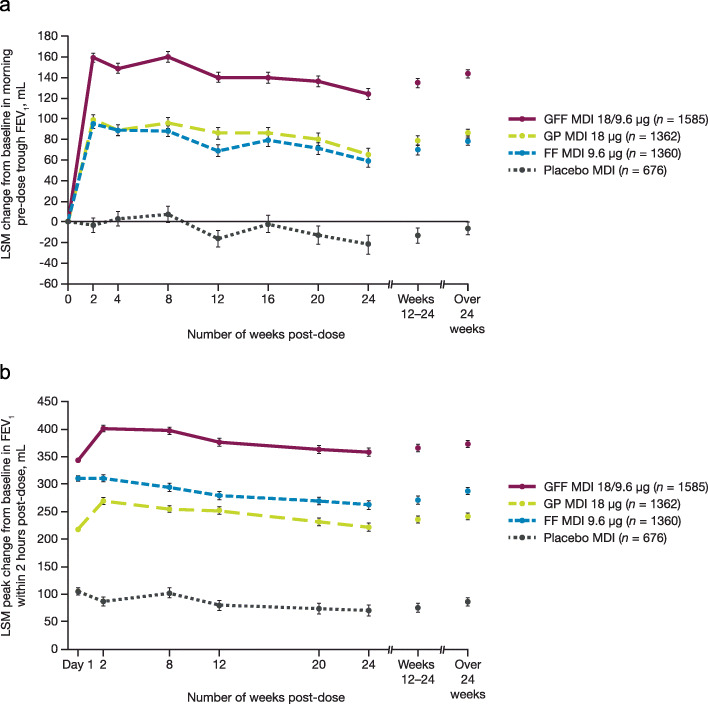
Lung function endpoints over time. Change from baseline in morning pre-dose trough FEV_1_ (**a**) and peak change from baseline in FEV_1_ within 2 h post-dose (**b**) (pooled ITT population). Data are least squares mean ± standard error. FEV_1_, forced expiratory volume in 1 s; FF, formoterol fumarate; GFF, glycopyrrolate/formoterol fumarate; GP, glycopyrrolate; ITT, intent-to-treat; LSM, least squares mean; MDI, metered dose inhaler

In the pooled ITT population, treatment with GFF MDI improved morning pre-dose trough FEV_1_ at week 24 versus GP MDI (least squares mean [LSM] difference [95% confidence interval (CI)]: 59 mL [43, 75 mL]), FF MDI (LSM difference [95% CI]: 65 mL [48, 81 mL]) and placebo MDI (LSM difference [95% CI]: 146 mL [125, 166 mL]), all *p* < 0.0001; Figs. 3a and 4a. These findings were consistent with the results for GFF MDI versus comparators in all three PINNACLE studies (Fig. 3a; all comparisons *p* ≤ 0.0003). The improvements with GFF MDI versus comparators in morning pre-dose trough FEV_1_ over time in the pooled ITT population further supported the results of the individual studies (Fig. 4a). In both the individual studies and the pooled ITT population, the treatment differences for GFF MDI versus placebo MDI consistently exceeded the minimal clinically important difference (MCID) threshold of 100 mL [12].

The treatment benefits with GFF MDI versus comparators observed over 24 weeks in the individual studies and pooled analysis were comparable to those observed at week 24 (all *p* < 0.0001; Additional file 1: Fig. S1(a)). GP MDI and FF MDI monotherapies consistently improved morning pre-dose trough FEV_1_ at week 24 and over 24 weeks compared to placebo MDI (all *p* ≤ 0.0138).

The peak change from baseline in FEV_1_ within 2 h post-dose at week 24 and over 24 weeks was significantly improved by GFF MDI versus monocomponents and placebo MDI in the pooled analysis as well as all three PINNACLE studies (all *p* < 0.0001; Figs. 3b, 4b, and Additional file 1: Fig. S1(b)). GP MDI and FF MDI also improved peak FEV_1_ at week 24 and over 24 weeks versus placebo MDI (all *p* < 0.0001).

#### Exacerbations

**Fig. 5:**
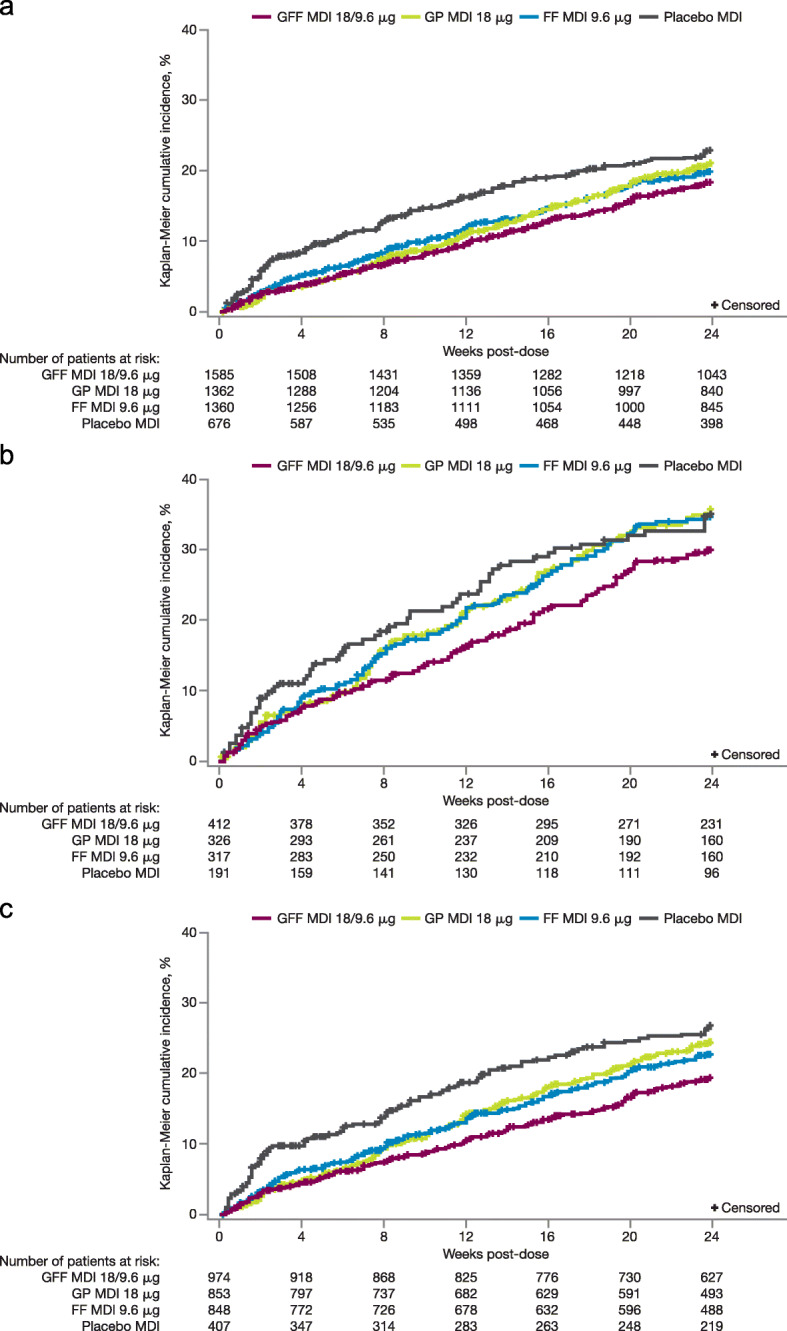
Time to first moderate or severe COPD exacerbation in the pooled ITT population (**a**), exacerbation history population (**b**), and CAT ≥15 population (**c**). The exacerbation history population includes patients with ≥1 moderate or severe exacerbations in the previous year. CAT, COPD assessment test; COPD, chronic obstructive pulmonary disease; FF, formoterol fumarate; GFF, glycopyrrolate/formoterol fumarate; GP, glycopyrrolate; ITT, intent-to-treat; MDI, metered dose inhaler.

In the pooled ITT population, the proportion of patients who experienced a moderate or severe COPD exacerbation in the GFF MDI treatment arm was 17.7%, compared with 20.2, 19.1, and 21.6% in the GP MDI, FF MDI, and placebo MDI groups, respectively (Additional file 1: Table S1). GFF MDI delayed the time to first moderate or severe COPD exacerbation compared with GP MDI, FF MDI, and placebo MDI, with the difference versus comparators maintained over the treatment period in the pooled ITT population, the exacerbation history subgroup and the symptomatic subgroup (Fig. 5).

**Fig. 6:**
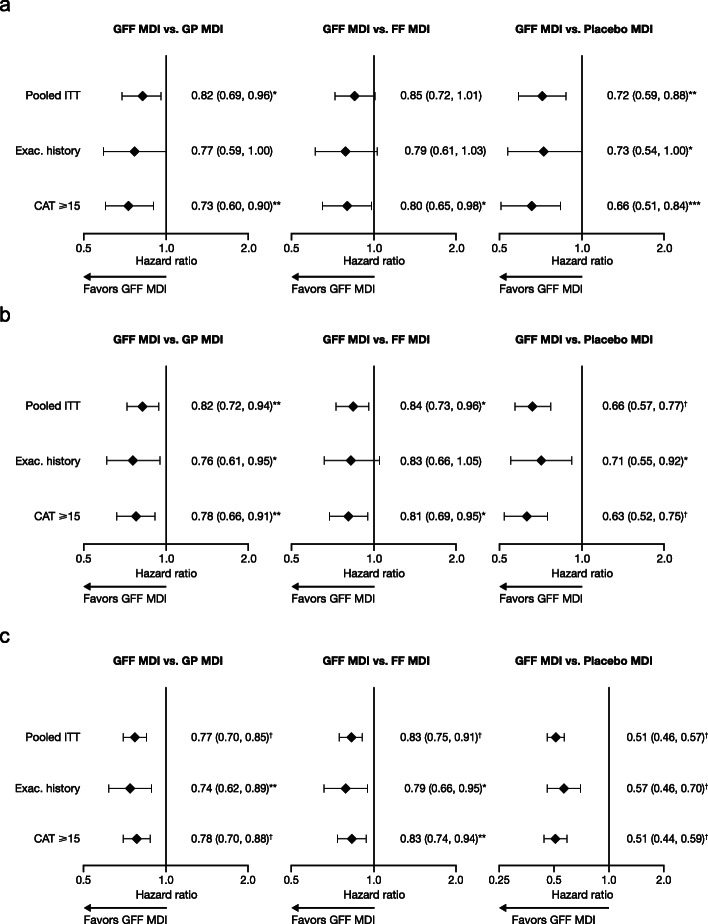
Time to first moderate or severe COPD exacerbation (**a**), time to treatment failure (**b**), and time to first CID (**c**) in the pooled ITT, exacerbation history, and CAT ≥15 populations. Data are hazard ratios (95% confidence intervals). ^*^p < 0.05; ^**^p < 0.01; ^***^p < 0.001; ^†^*p* < 0.0001. The exacerbation history population includes patients with ≥1 moderate or severe exacerbations in the previous year. CAT, COPD assessment test; CID, clinically important deterioration; COPD, chronic obstructive pulmonary disease; Exac., exacerbation; FF, formoterol fumarate; GFF, glycopyrrolate/formoterol fumarate; GP, glycopyrrolate; ITT, intent-to-treat; MDI, metered dose inhaler.

GFF MDI reduced the risk of a moderate or severe COPD exacerbation compared with GP MDI (hazard ratio [HR] [95% CI] 0.82 [0.69, 0.96]; *p* = 0.0168), FF MDI (HR [95% CI] 0.85 [0.72, 1.01]; *p* = 0.0628) and placebo MDI (HR [95% CI] 0.72 [0.59, 0.88]; *p* = 0.0012; Fig. 6a). Compared with the pooled ITT population, the proportions of patients who experienced a moderate or severe COPD exacerbation were slightly larger in the symptomatic subgroup (GFF MDI [18.7%], GP MDI [23.3%], FF MDI [21.5%], and placebo MDI [24.8%]), and as expected were noticeably larger in the exacerbation history subgroup (GFF MDI [28.9%], GP MDI [33.4%], FF MDI [32.8%], and placebo MDI [33.0%]; Additional file 1: Table S1). In general, the magnitude of treatment benefit in reducing the risk of a moderate or severe COPD exacerbation was greater with GFF MDI versus comparators in the exacerbation history and symptomatic subgroups than the pooled ITT population, as indicated by smaller hazard ratios (Fig. 6a).

The annualized rate of moderate or severe exacerbations was also numerically lower with GFF MDI versus monocomponents and placebo MDI in all the analysis populations (Additional file 1: Table S1).

#### Treatment failure

Treatment failure with GFF MDI was observed in 27.6% of patients (*n* = 438), compared with 31.6% of patients with GP MDI (*n* = 431), 30.6% with FF MDI (*n* = 416), and 36.8% with placebo MDI (*n* = 249; Additional file 1: Table S2). In the pooled ITT population, GFF MDI reduced the risk of treatment failure by 18% (*p* = 0.0043), 16% (*p* = 0.0107), and 34% (*p* < 0.0001) versus GP MDI, FF MDI, and placebo MDI, respectively (Fig. 6b). In line with the findings for exacerbations, the exacerbation history and symptomatic subgroups generally experienced similar or larger reductions in the risk of treatment failure with GFF MDI versus comparators than the pooled ITT population (Fig. 6b, Additional file 1: Table S2).

#### CID

A CID event occurred in 58.4% of patients (*n* = 925) treated with GFF MDI, compared with 66.0% (*n* = 899), 62.1% (*n* = 845), and 74.4% (*n* = 503) of patients who received GP MDI, FF MDI, and placebo MDI, respectively (Additional file 1: Table S2). GFF MDI reduced the risk of CID versus GP MDI, FF MDI, and placebo MDI in the pooled ITT population (23, 17, and 49% reductions, respectively; all *p* < 0.0001), with similar effects observed in the exacerbation history and symptomatic subgroups (all *p* < 0.05) (Fig. 6c, Additional file 1: Table S2).

### Safety

Overall, the incidences of treatment-emergent adverse events (TEAEs), drug-related TEAEs, TEAEs leading to early discontinuation, and serious TEAEs were similar across treatment groups (Table 2). Most TEAEs were mild to moderate in severity, with severity comparable across treatment groups. The most commonly reported TEAEs overall were upper respiratory tract infection, viral upper respiratory tract infection, and cough (4.8, 4.7, and 2.7%, respectively in the pooled population, and 4.4, 4.7 and 3.4%, respectively in the GFF MDI group) (Table 2).

**Table 2:** Summary of safety data (pooled safety population)

	GFF MDI 18/9.6 μg (*n* = 1588)	GP MDI 18 μg (*n* = 1364)	FF MDI 9.6 μg (*n* = 1370)	Placebo MDI (*n* = 678)	All patients (*n* = 5000)
TEAEs, *n* (%) [number of events]					
≥ 1 TEAE	923 (58.1) [2317]	750 (55.0) [1946]	762 (55.6) [1866]	386 (56.9) [921]	2821 (56.4) [7050]
Mild	396 (24.9)	317 (23.2)	348 (25.4)	174 (25.7)	1235 (24.7)
Moderate	404 (25.4)	326 (23.9)	308 (22.5)	162 (23.9)	1200 (24.0)
Severe	123 (7.7)	107 (7.8)	106 (7.7)	50 (7.4)	386 (7.7)
Drug-related^a^ TEAEs	172 (10.8) [276]	150 (11.0) [256]	144 (10.5) [228]	69 (10.2) [115]	535 (10.7) [875]
Serious TEAEs	133 (8.4) [171]	107 (7.8) [155]	106 (7.7) [129]	50 (7.4) [65]	396 (7.9) [520]
Drug-related^a^ serious TEAEs	10 (0.6) [11]	15 (1.1) [21]	8 (0.6) [9]	3 (0.4) [3]	36 (0.7) [44]
TEAEs leading to early discontinuation	91 (5.7) [125]	80 (5.9) [133]	71 (5.2) [120]	43 (6.3) [56]	285 (5.7) [434]
Deaths (all causes)					
Treatment period	5 (0.3)	1 (0.1)	2 (0.1)	2 (0.3)	10 (0.2)
Treatment period + 14 days	6 (0.4)	2 (0.1)	2 (0.1)	2 (0.3)	12 (0.2)
TEAEs occurring in ≥2% of patients in any treatment arm, preferred term, *n* (%)					
Upper respiratory tract infection	70 (4.4)	67 (4.9)	59 (4.3)	42 (6.2)	238 (4.8)
Viral upper respiratory tract infection	75 (4.7)	61 (4.5)	71 (5.2)	26 (3.8)	233 (4.7)
Cough	54 (3.4)	37 (2.7)	32 (2.3)	14 (2.1)	137 (2.7)
COPD^b^	40 (2.5)	42 (3.1)	30 (2.2)	20 (2.9)	132 (2.6)
Dyspnea	35 (2.2)	32 (2.3)	35 (2.6)	26 (3.8)	128 (2.6)
Nasopharyngitis	45 (2.8)	25 (1.8)	29 (2.1)	19 (2.8)	118 (2.4)
Sinusitis	28 (1.8)	27 (2.0)	33 (2.4)	16 (2.4)	104 (2.1)
Headache	30 (1.9)	31 (2.3)	35 (2.6)	7 (1.0)	103 (2.1)
Back pain	36 (2.3)	29 (2.1)	25 (1.8)	11 (1.6)	101 (2.0)
Bronchitis	24 (1.5)	35 (2.6)	18 (1.3)	17 (2.5)	94 (1.9)
Hypertension	28 (1.8)	20 (1.5)	21 (1.5)	24 (3.5)	93 (1.9)
Pneumonia	28 (1.8)	24 (1.8)	18 (1.3)	17 (2.5)	87 (1.7)
Urinary tract infection	33 (2.1)	21 (1.5)	14 (1.0)	12 (1.8)	80 (1.6)

^a^Possibly, probably, or definitely related to study drug, as per investigator’s judgment prior to unblinding. ^b^Worsening of COPD defined as a COPD exacerbation since the patient’s last visit. COPD exacerbations were only recorded as an AE if they were considered to be a serious TEAE

*COPD* chronic obstructive pulmonary disease, *FF* formoterol fumarate, *GFF* glycopyrrolate/formoterol fumarate, *GP* glycopyrrolate, *MDI* metered dose inhaler, *TEAE* treatment-emergent adverse event

A total of 12 treatment-emergent deaths were reported across the three pivotal Phase III 24-week studies. None of the deaths were considered by the investigator to be related to the study drug, and two occurred within 14 days post-treatment. A total of 6 patients in the GFF MDI group (0.4%) and 2 patients in each of the GP MDI, FF MDI, and placebo MDI groups (0.1–0.3%) experienced a fatal event. Six deaths had a probable cardiovascular cause (GFF MDI, *n* = 3; GP MDI, *n* = 1; FF MDI, *n* = 2); none had a probable respiratory cause (full details are provided in Additional file 1: File A1). Overall, the pooled safety analysis identified no new safety concerns among the most commonly-reported TEAEs, and no clinically meaningful differences were observed between treatment groups.

## Discussion

This pooled analysis of data from PINNACLE-1, -2, and -4 illustrates consistent lung function benefits across the individual studies, with improvements in the change from baseline in morning pre-dose trough FEV_1_ at week 24 and over 24 weeks with GFF MDI versus placebo MDI exceeding the MCID of 100 mL [12]. GFF MDI also consistently provided significant improvements in trough FEV_1_ and peak change from baseline in FEV_1_ within 2 h post-dose compared with GP MDI and FF MDI [7, 9]. Improvements in lung function in the PINNACLE studies were generally similar to those reported in other pivotal studies of LAMA/LABA FDCs [10, 11, 13]. A network meta-analysis performed using all available data of fixed-dose LAMA/LABA combinations (including the PINNACLE studies) also found that GFF MDI had comparable efficacy to other LAMA/LABAs in improving lung function [14].

Pooling data across the three pivotal PINNACLE studies allowed for assessment of study endpoints, which were not well powered in the individual studies, such as differential treatment effects on COPD exacerbations. Many studies assessing COPD symptom and exacerbation endpoints enroll patients with a high symptom burden or exacerbation history [10, 11, 15] to observe more substantial treatment effects. Notably, even though the PINNACLE studies did not enrich the patient population for these characteristics, clinically meaningful reductions in the risk of a moderate or severe COPD exacerbation, treatment failure, and CID were observed with GFF MDI compared with GP MDI, FF MDI, and placebo MDI in the pooled ITT population.

Given that the inclusion criteria of the PINNACLE studies did not enrich the study population for patients with a high symptom burden or exacerbation history, the integrated analyses included subgroups that examined the impact of two risk factors for exacerbations (high symptom burden [CAT score ≥15] and/or a recent exacerbation history [≥1 moderate or severe exacerbations in the past year]) on treatment benefit. While the GOLD report uses a cut-off of CAT ≥10 to define symptomatic patients [1], this pooled analysis defined the symptomatic population as CAT ≥15 based on the threshold pre-specified in the PINNACLE-4 analysis plan. Previous pooled findings from PINNACLE-1 and -2 have shown that results for CAT ≥10 and CAT ≥15 subgroups were generally similar [6]. Exacerbation reductions for GFF MDI versus monocomponents were greater in symptomatic patients and those with a previous exacerbation history compared to the pooled ITT population (as indicated by smaller hazard ratios), suggesting that these subgroups of patients may derive an even larger benefit from LAMA/LABA treatment compared with monotherapies. The pooled ITT and symptomatic populations experienced similar treatment benefits with regard to the time to first CID. However, this was not unexpected considering that the time to first CID was primarily driven by the ≥100 mL decline in morning pre-dose trough FEV_1,_ and a previous analysis of pooled data from PINNACLE-1 and -2 showed that the magnitude of improvement in the morning pre-dose trough FEV_1_ was independent of patient baseline CAT scores [6], and would also not be expected to relate to exacerbation history.

The exacerbations treatment pathway described in the GOLD 2020 Report recommends dual LAMA/LABA combination therapy for patients in whom an individual LAMA or LABA is not adequately controlling COPD exacerbations unless they have a blood eosinophil count ≥300 cells/μL or a high risk of further exacerbations [1]. The decreased risk of exacerbations demonstrated with GFF MDI relative to GP MDI and FF MDI in patients with moderate-to-very severe COPD from the PINNACLE studies supports this treatment recommendation. However, the comparable safety profile between GFF MDI, GP MDI and FF MDI, and the fact that treatment differences in exacerbation risk were observed even in a population including low-risk patients, supports the use of dual LAMA/LABA therapy regardless of patients’ symptom burden and exacerbation history in order to reduce the chance of experiencing an exacerbation that could substantially impact their lung function and quality of life [2, 3].

The PINNACLE studies also assessed dyspnea using the transition dyspnea index (TDI) scale [9, 16]. However, it was not feasible to pool these data, as PINNACLE-1 and -2 used a self-administered computerized (SAC) version of the tool, while PINNACLE-4 used the more established paper interviewer-administered version, and thus this endpoint was not included in the pooled analysis. The SAC TDI is reported on a continuous scale, while the interviewer-administered TDI is on an integer Likert scale [17–19]. All three PINNACLE studies assessed quality of life using the SGRQ [7, 9], although a pooled analysis was not considered useful due to variation in the findings between PINNACLE-1 and -2 versus PINNACLE-4. The changes from baseline in SGRQ total score at week 24 with all active treatments were greater in PINNACLE-4 than in PINNACLE-1 and -2 [7, 9], which may relate to the geographic difference in the populations studied and the modifying effect of socioeconomic status on quality of life outcomes [20].

The analysis of pooled safety data enabled the safety and tolerability of GFF MDI to be investigated in a much larger patient population than any of the individual studies alone. The results in the pooled population were commensurate with the individual studies [7, 9], and demonstrated that all treatments were well tolerated, with no unexpected safety signals identified.

Limitations of the analyses include the fact that the three individual studies were produced at different times and, accordingly, some of the pooled analyses were specified after results from PINNACLE-1 and -2 were available (but before the unblinding of PINNACLE-4 data). It should be noted that GFF MDI and the comparators used in the PINNACLE studies were all formulated using co-suspension delivery technology (other than tiotropium in PINNACLE-1). Hence, these studies could not assess the potential impact of the co-suspension delivery technology on clinical outcomes.

While many studies that evaluate COPD exacerbations are conducted over 52 weeks [15, 21, 22], the PINNACLE studies were 24 weeks in duration. However, due to the large number of patients (*n* = 4983) and patient-years of exposure (> 2000), the fact that patients were recruited across all four seasons of the year in each study, and the use of time-to-event analyses to handle censoring due to study discontinuation following a COPD exacerbation, this pooled analysis provides a compelling body of evidence for the benefit of GFF MDI in preventing exacerbations.

## Conclusions

Pooled analyses of lung function endpoints (pre-dose trough FEV_1_ and peak FEV_1_) from the PINNACLE studies supported the findings from the individual studies, with GFF MDI 18/9.6 μg demonstrating significant, clinically meaningful improvements versus placebo and monocomponents in patients with moderate-to-very severe COPD. GFF MDI reduced the risk of moderate or severe COPD exacerbations, CID, and treatment failure compared with monocomponents and placebo MDI, even in a patient population that was not enriched for symptoms or exacerbation history. Treatment benefits for GFF MDI versus monotherapies on exacerbation risk were generally even greater in subgroups of patients with higher symptom burden (CAT score ≥15) and those with an exacerbation history (≥1 moderate or severe exacerbation in the past year), providing additional support for the importance of LAMA/LABA therapy in these patient populations.

## Supplementary information

**Additional file: 1 File S1.** Results – safety. **Table S1.** Moderate or severe COPD annualized exacerbations rate (pooled ITT, exacerbation history, and CAT ≥15 populations). **Table S2.** Patients with treatment failure or CID (pooled ITT, exacerbation history, and CAT ≥15 populations). **Figure S1.** Lung function endpoints over 24 weeks. Change from baseline in morning pre-dose trough FEV_1_ (A) and peak change from baseline in FEV_1_ within 2 h post-dose (B) (ITT population of the individual and pooled studies).

## Data Availability

The datasets used and analyzed during the current study are available on reasonable request in accordance with AstraZeneca’s data sharing policy described at https://astrazenecagrouptrials.pharmacm.com/ST/Submission/Disclosure.

## Abbreviations

CAT: COPD assessment test
CI: Confidence interval
CID: Clinically important deterioration
COPD: Chronic obstructive pulmonary disease
DPI: Dry powder inhaler
Exac: Exacerbation
FDC: Fixed-dose combination
FEV_1_: Forced expiratory volume in 1 s
FF: Formoterol fumarate
FVC: Forced vital capacity
GFF: Glycopyrrolate/formoterol fumarate
GOLD: Global Initiative for Chronic Obstructive Lung Disease
GP: Glycopyrrolate
HR: Hazard ratio
ICS: Inhaled corticosteroid
ITT: Intent-to-treat
LABA: Long-acting β_2_-agonist
LAMA: Long-acting muscarinic antagonist
LSM: Least squares mean
MCID: Minimum clinically important difference
MDI: Metered dose inhaler
NA: Not applicable
SAC: Self-administered computerized
SD: Standard deviation
SGRQ: St. George’s Respiratory Questionnaire
TDI: Transition dyspnea index
TEAE: Treatment-emergent adverse event
