# Supplementary material for: Benefits of glycopyrrolate/formoterol fumarate metered dose inhaler (GFF MDI) in improving lung function and reducing exacerbations in patients with moderate-to-very severe COPD: a pooled analysis of the PINNACLE studies
Source: Respir Res. 2020 May 25;21:128. doi: 10.1186/s12931-020-01388-y (PMC7249639; doi:10.1186/s12931-020-01388-y)
Supplement: Supplementary file 1 — Additional file: 1 File S1. Results – safety. Table S1. Moderate or severe COPD annualized exacerbations rate (pooled ITT, exacerbation history, and CAT ≥15 populations). Table S2. Patients with treatment failure or CID (pooled ITT, exacerbation history, and CAT ≥15 populations). Figure S1. Lung function endpoints over 24 weeks. Change from baseline in morning pre-dose trough FEV1 (A) and peak change from baseline in FEV1 within 2 h post-dose (B) (ITT population of the individual and pooled studies). [file 12931_2020_1388_MOESM1_ESM.docx]

# Additional file 1

**File A1.** Results – Safety

Twelve deaths were reported in the pooled safety population, with the following causes of death: cardiac arrest, metastatic neoplasm, fatal gunshot wound, acute myocardial infarction, cardiovascular death of unknown cause, metastatic lung cancer (GFF MDI); aspiration pneumonia, hemorrhagic stroke (GP MDI); peripheral artery thrombosis, hypoglycemic coma (FF MDI); unknown cause of death, metastatic lung cancer (placebo MDI). The deaths resulting from metastatic neoplasm, acute myocardial infarction, and aspiration pneumonia occurred in the post-treatment period.

**Table A1.** Moderate or severe COPD annualized exacerbations rate (pooled ITT, exacerbation history, and CAT ≥15 populations)

|  | **GFF MDI**  **18/9.6 µg** | **GP MDI**  **18 µg** | **FF MDI**  **9.6 µg** | **Placebo MDI** |
| --- | --- | --- | --- | --- |
| **Patients with moderate or severe COPD exacerbations, n (%)** | | | | |
| Pooled ITT^a^ | 281 (17.7) | 275 (20.2) | 260 (19.1) | 146 (21.6) |
| Exac. history^b^ | 119 (28.9) | 109 (33.4) | 104 (32.8) | 63 (33.0) |
| CAT ≥15^c^ | 182 (18.7) | 199 (23.3) | 182 (21.5) | 101 (24.8) |
| **Adjusted rate of moderate or severe COPD exacerbations, per year** | | | | |
| Pooled ITT^a^ | 0.48 | 0.55 | 0.54 | 0.66 |
| *Rate ratio for GFF MDI vs. comparators (95% CI)* | – | 0.88  (0.73, 1.05) | 0.89  (0.75, 1.07) | 0.74  (0.59, 0.92)^**^ |
| Exac. history^b^ | 0.96 | 1.15 | 1.02 | 1.21 |
| *Rate ratio for GFF MDI vs. comparators (95% CI)* | – | 0.83  (0.63, 1.10) | 0.94  (0.71, 1.25) | 0.79  (0.57, 1.09) |
| CAT ≥15^c^ | 0.52 | 0.66 | 0.62 | 0.77 |
| *Rate ratio for GFF MDI vs. comparators (95% CI)* | – | 0.80  (0.64, 0.98)^*^ | 0.84  (0.68, 1.04) | 0.68  (0.53, 0.88)^**^ |

^a^GFF MDI (*n* = 1585), GP MDI (*n* = 1362), FF MDI (*n* = 1360), Placebo MDI (*n* = 676).

^b^GFF MDI (*n* = 412), GP MDI (*n* = 326), FF MDI (*n* = 317), Placebo MDI (*n* = 191).

^c^GFF MDI (*n* = 974), GP MDI (*n* = 853), FF MDI (*n* = 848), Placebo MDI (*n* = 407).

^*^*p* < 0.05; ^**^*p* < 0.01

CAT, COPD assessment test; CI, confidence interval; COPD, chronic obstructive pulmonary disease; Exac., exacerbation; FF, formoterol fumarate; GFF, glycopyrrolate/formoterol fumarate; GP, glycopyrrolate; ITT, intent-to-treat; MDI, metered dose inhaler.

**Table A2.** Patients with treatment failure or CID (pooled ITT, exacerbation history, and CAT ≥15 populations)

|  | **GFF MDI 18/9.6 µg** | **GP MDI**  **18 µg** | **FF MDI**  **9.6 µg** | **Placebo MDI** |
| --- | --- | --- | --- | --- |
| **Patients with treatment failure, n (%)** | | | | |
| Pooled ITT^a^ | 438 (27.6) | 431 (31.6) | 416 (30.6) | 249 (36.8) |
| Exac. history^b^ | 162 (39.3) | 151 (46.3) | 135 (42.6) | 89 (46.6) |
| CAT ≥15^c^ | 294 (30.2) | 307 (36.0) | 292 (34.4) | 171 (42.0) |
| **Patients with CID, n (%)** |  |  |  |  |
| Pooled ITT^a^ | 925 (58.4) | 899 (66.0) | 845 (62.1) | 503 (74.4) |
| Exac. history^b^ | 251 (60.9) | 230 (70.6) | 213 (67.2) | 147 (77.0) |
| CAT ≥15^c^ | 576 (59.1) | 559 (65.5) | 528 (62.3) | 304 (74.7) |

^a^GFF MDI (*n* = 1585), GP MDI (*n* = 1362), FF MDI (*n* = 1360), Placebo MDI (*n* = 676).

^b^GFF MDI (*n* = 412), GP MDI (*n* = 326), FF MDI (*n* = 317), Placebo MDI (*n* = 191).

^c^GFF MDI (*n* = 974), GP MDI (*n* = 853), FF MDI (*n* = 848), Placebo MDI (*n* = 407).

CAT, COPD assessment test; CID, clinically important deterioration; Exac., exacerbation; FF, formoterol fumarate; GFF, glycopyrrolate/formoterol fumarate; GP, glycopyrrolate; ITT, intent-to-treat; MDI, metered dose inhaler.

**Fig. A1** Lung function endpoints over 24 weeks. Change from baseline in morning pre-dose trough FEV_1_ (A) and peak change from baseline in FEV_1_ within 2 hours post-dose (B) (ITT population of the individual and pooled studies)


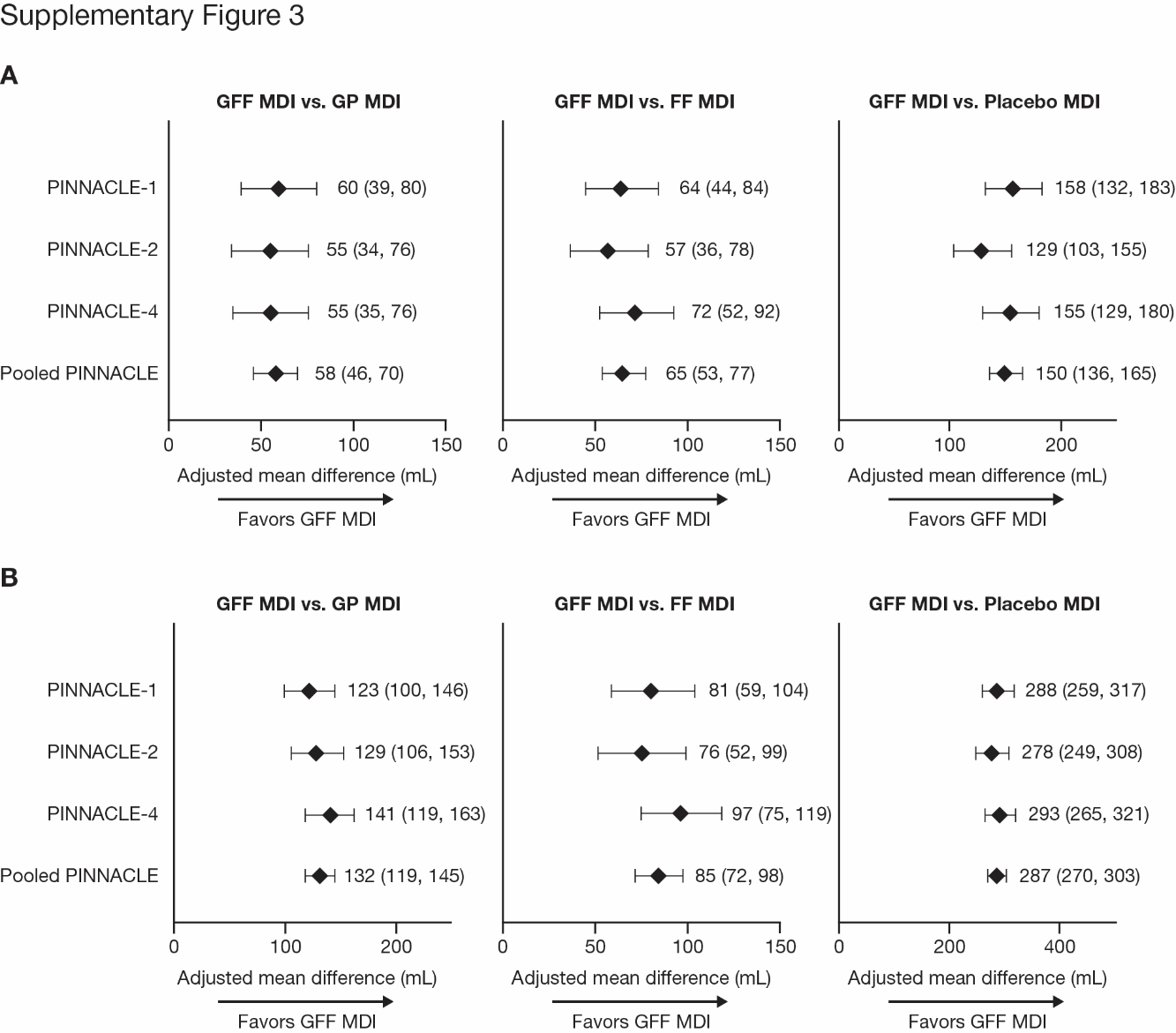


Data are least squares mean treatment differences (95% confidence intervals), shown in mL.
*p* < 0.0001 for all comparisons.

FEV_1_, forced expiratory volume in 1 second; FF, formoterol fumarate; GFF, glycopyrrolate/formoterol fumarate; GP, glycopyrrolate; ITT, intent-to-treat; MDI, metered dose inhaler.
